# Using electronic health records to evaluate a children and young people’s social prescribing service: challenges and implications for research and practice

**DOI:** 10.1136/bmjment-2025-302442

**Published:** 2026-04-24

**Authors:** Jessica K Bone, Feifei Bu, Daisy Fancourt, Daniel Hayes

**Affiliations:** 1 Research Department of Behavioural Science and Health, Institute of Epidemiology & Health Care, University College London, London, UK

**Keywords:** Adolescent, Psychosocial Intervention, Mental Health

## Abstract

**Background:**

Preliminary evidence indicates that social prescribing (SP) can improve children and young people’s (CYP) well-being but is limited by small non-representative samples and often relies on descriptive statistics. Given the wide implementation of SP in the UK, administrative records provide a unique opportunity to understand current practice and assess impacts on well-being.

**Objectives:**

(1) To understand the quality of data captured in SP administrative records. (2) To explore which CYP are currently receiving SP and what SP entails in practice. (3) To assess the impact of SP on well-being.

**Methods:**

We used administrative records from one CYP SP service in England. Records were extracted from Joy, an online platform for managing SP. Over 18 months, 770 age-eligible CYP were referred to SP, 203 of whom were successfully discharged and completed two pre-post measures of well-being (the short Warwick-Edinburgh Mental Wellbeing Scale; SWEMWBS) at least 7 days apart. We used descriptive statistics, a paired t-test to assess changes in well-being and linear regressions with interactions to test effect modification.

**Findings:**

Missing data was the largest issue, with ethnicity missing for 94% of CYP. A lack of detail and inconsistent recording for both individual characteristics and SP practices also presented challenges. Despite this, we identified that most CYP were referred by their GP, followed by their school, with 97% referred because of their mental health. The most common pathway was to receive SP for around 90 days, with 10–15 link worker contacts and 6 contact hours. Following SP, SWEMWBS scores improved by 3.72 points (t(202)=17.50, 95% CI 3.30 to 4.14, p<0.001), a 20% relative increase. Exploratory analyses suggested that this increase was greater for those with lower baseline well-being.

**Conclusions:**

Despite numerous challenges with missing data and data quality, we found that CYP well-being increases following SP (as it is currently implemented). The moderate effect size was consistent with larger studies of adults.

**Clinical implications:**

Further development of online platforms is needed to monitor access to, nature of and efficacy of SP. For those working in SP, we recommend more training, implementation of standardised guidelines and designated time to update records.

**WHAT IS ALREADY KNOWN ON THIS TOPIC**

- Social prescribing (SP) has been widely implemented in the UK, but there is very little evidence on what it looks like or whether it works for children and young people (CYP).

**WHAT THIS STUDY ADDS**

- We show that it is feasible to evaluate SP for CYP using administrative records and identify several priorities for improving data quality.
- We describe the typical SP pathway in one CYP’s service in England, which includes receiving SP for around 90 days, with 10–15 link worker contacts and 6 contact hours.
- We found evidence for clinically significant improvements in CYP’s well-being from their first to their last SP session.

**HOW THIS STUDY MIGHT AFFECT RESEARCH, PRACTICE OR POLICY**

- To meet best practice guidelines for recording and evaluating SP, we need further collaboration between online platform providers and SP services, more training and time for link workers, and for research and policy to emphasise the importance of measuring outcomes.

## Background

 Social prescribing (SP) is a care pathway that aims to connect people with non-medical forms of support within their community, based on their values and preferences.^1^ It aims to address people’s social, emotional and practical needs, which are often closely related to their medical needs but not routinely addressed by clinical treatments. In the UK, SP typically consists of referral to a link worker, or other similar professional, who works with the individual to develop a personalised care plan that connects them to community support. This may include a wide range of activities such as sports, arts, culture, volunteering, counselling, housing support, training and employment advice.^2^

SP could reduce the impact of social inequalities on children and young people (CYP), with potential for sustained benefits throughout the life course.^3^ Yet, despite being funded as an all-age approach in the UK,^4^ SP research, policy and practice have predominantly focused on adults.^3 5^ This is particularly problematic because SP approaches for adults may not be suitable for CYP, who have different social contexts^6^ and face different challenges, including 75% of mental health problems emerging during adolescence.^7^ Additionally, CYP and adults may access SP through differing routes in the UK. Unlike adults who are primarily referred by General Practitioners (GPs), CYP may access SP via alternative referral routes, including educational institutions and social care.^5^

Preliminary evidence among CYP is promising, with six uncontrolled pre-post quantitative studies^8^,^13^ and one randomised controlled trial (RCT)^14^ reporting improved well-being, reduced loneliness and less healthcare utilisation following SP. But only one of these included over 100 participants in outcome analyses^14^ (n=6 to n=77 in the others),^8^,^13^ meaning most were likely underpowered. A scoping review concluded that research with CYP has been further limited by not being representative of the target population, not including control groups, short follow-ups, attrition, limited use of standardised measures and statistical analyses, and unclear reporting.^3^

Large RCTs of SP for CYP are clearly needed, and several are underway.^15 16^ But existing data capturing the widespread implementation of SP are also important.^5^ Administrative data are uniquely placed to evaluate real-world heterogeneity, allowing us to explore how CYP are currently accessing SP, whether there are inequalities in who receives SP, and whether SP (as currently implemented) can improve well-being. Link workers in the UK often use electronic health systems like EMIS or SystmOne, but many use bespoke systems designed for SP,^17^ including Joy, Elemental and Social Rx Connect. These platforms allow link workers to monitor referrals, connect with local services, record appointments and prescriptions, and measure impact. These platforms only include individuals who receive SP, meaning no control group is available for comparative analyses. However, they capture non-primary care referrals, provide rich data on interventions and measure outcomes, providing major advantages over GP records. A growing body of research using administrative records to evaluate SP has emerged,^218^,^22^ but no studies have focused on CYP.

### Objectives

We had three objectives. First, to understand the quality of administrative data captured in a platform for SP with CYP. Second, to explore which CYP are receiving SP, and what SP looks like for them. Finally, to assess the impact of SP on CYP well-being. Findings were used to make recommendations on how electronic health records should capture SP and identify targets for improving practice.

## Methods

### Data

We used administrative data from one SP service for CYP aged 11–18 years (or up to 25 with special educational needs and disabilities) in one county in North East England. This county includes diverse areas, with 5–10% of the population living in areas in the most deprived Index of Multiple Deprivation (IMD) decile and approximately 20% in the least deprived decile.^23^ At the time of the evaluation, the CYP SP service employed seven Social Prescribers, including a Lead CYP Social Prescriber and two Senior Social Prescribers. All Social Prescribers completed extensive training on neurodiversity, making every contact count, personalised care, physical, mental and emotional well-being, safeguarding, self-harm and suicide awareness, and domestic abuse, among other areas. The service was commissioned to support many aspects of health and well-being, including mental health, but it did not specialise in mental health.

The service used Joy,^24^ an online platform for healthcare professionals to manage SP, monitor clients, connect with local services and measure impact. Joy is used in 30% of primary care networks, with 25 000 patients referred through Joy each month, and >19 million patients to date.^24^ Administrators and link workers used Joy to record referral sources and reasons, demographic information, contacts with individuals, prescribed activities and services, outcomes, discharge and case notes. Information is entered through a combination of dropdown lists and free text boxes.

**Figure 1:**
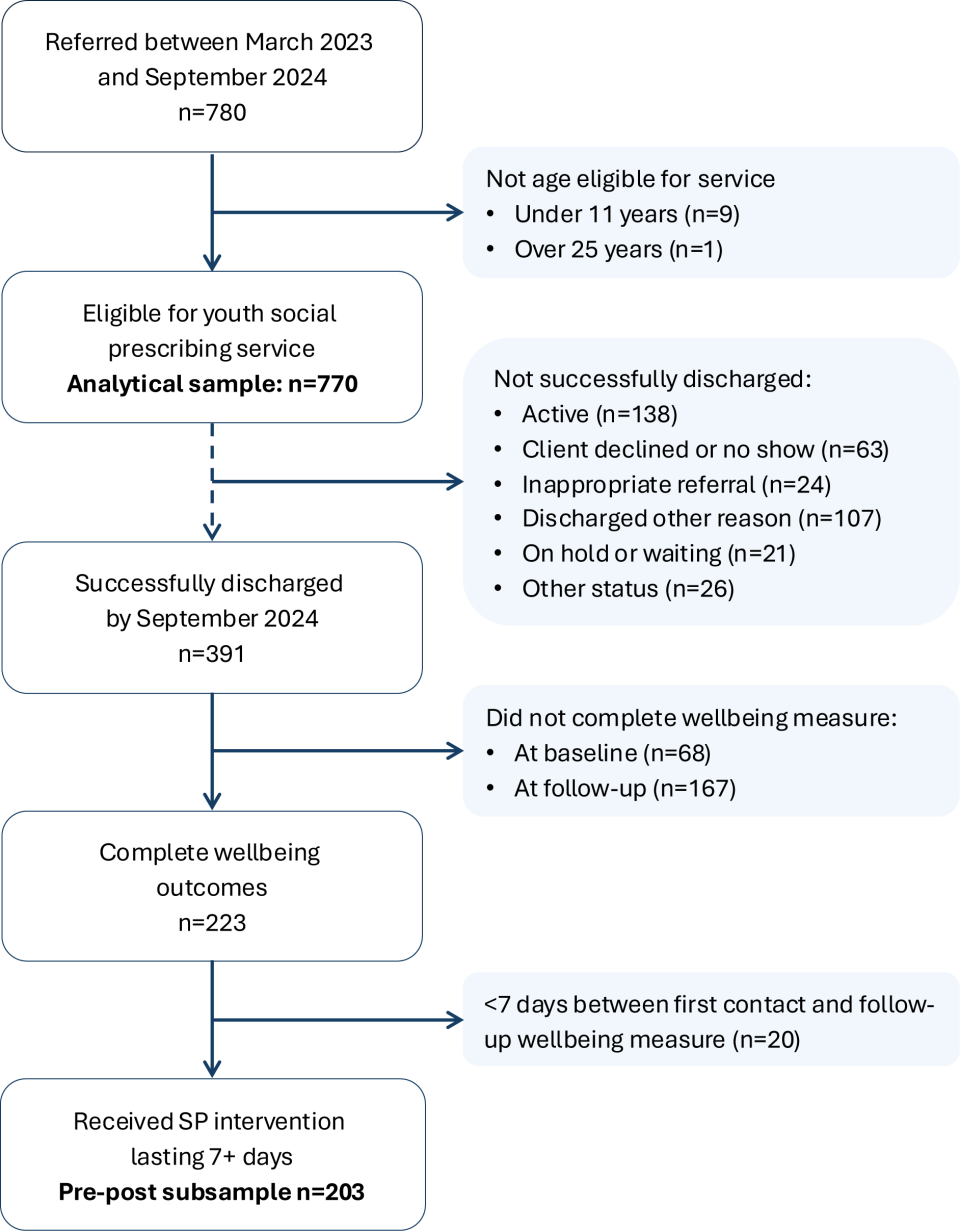
Flowchart showing sample selection. SP, social prescribing.

Data were available from Joy for March 2023 to September 2024. In this period, a total of 780 people were referred to SP. Of these, 770 were age-eligible for SP, and formed our analytical sample, in which we explored the characteristics of young people referred to SP. This included 391 CYP who had been successfully discharged, as well as 379 cases that were active (n=138), had declined SP (n=63), were inappropriate referrals (n=24), discharged early (n=107), waiting for SP (n=21) or had other status (n=26; figure 1).

We conducted further analyses among a subsample with data on well-being before and after receiving SP. To be included, individuals had to be successfully discharged from the SP service by September 2024 and have completed two measures of well-being. These outcome measures were typically completed at the first SP session and the final session before discharge. We also required that the final measure of well-being was completed at least 7 days after the first well-being measure, ensuring that all individuals received at least one SP session and maximising sample size. Of the 770 eligible for SP, 391 were successfully discharged, 223 of whom had complete well-being outcomes, and 203 received an SP intervention lasting 7 or more days. Our pre-post sample thus included 203 people, which had over 90% power to detect a mean difference of one SD in well-being (based on UK population norms)^25^ using a paired t-test with alpha=0.05. The average length of time between well-being measures was 89.7 days (SD=56.9).

### Measures

#### Individual characteristics

Administrators recorded clients’ age, gender and ethnicity based on their referral forms. We could not use gender and ethnicity due to the large proportion of missing data (92–94% missing; online supplemental table S1).

Young people were referred to SP from a range of sources, recorded using free text, which we collapsed into five categories: GP, school, other medical services (eg, A&E, public health nursing services), youth services or self-referral. Reasons for referral were recorded using >140 options, which we categorised into eight domains: 1) mental health, 2) physical health and well-being, 3) social relationships, 4) family issues, 5) lifestyle, 6) education, employment and training, 7) practical support and 8) other reasons (eg, abuse, gender identity; online supplemental table S2). Following previous analyses of SP records,^2^ grouping was done independently by two authors with disagreements (10%) discussed and resolved by consensus. Multiple referral reasons were allowed.

#### Well-being

The short Warwick-Edinburgh Mental Wellbeing Scale (SWEMWBS) measured mental well-being, with raw scores transformed to metric scores on the interval scale. Higher scores indicated greater well-being (range 7–35).^25^ The SWEMWBS was chosen by the SP service and StreetGames (a UK charity that ensures decisions are shaped by lived experience) when the service was originally established because it has been validated for ages 15–21,^26^ and the longer WEMWBS for ages 13 and above.^27^ As some individuals did not understand specific words in the SWEMWBS (eg, optimistic) or the rating scale, the SP service adapted it by adding emojis representing response options. It was completed with the link worker, who asked and explained the questions and recorded responses. The SWEMWBS was typically done in CYP’s first SP session (baseline), although sometimes there was a delay in completing it due to the waiting list, room availability or the CYP’s willingness to engage. However, we were not able to distinguish between each individual’s initial contacts with a link worker (eg, to arrange an appointment) versus their first SP session, so could not determine which SP session the SWEMWBS was completed in. The same process was then repeated in CYP’s final session before discharge (follow-up).

#### Social prescribing

Link workers recorded the date of their first contact with the client, which could be an introductory appointment, text or phone call. A first contact form was available for use at the first link worker appointment, but it was only completed for 36% (n=140) of individuals who were successfully discharged, as most link workers preferred to embed responses in staff notes instead. For these analyses, we did not have access to staff notes. We therefore calculated the length of the SP period as the number of days from the first recorded contact to the final SWEMWBS completion (which should be done during the final session before discharge). There were a few outliers in the length of SP, whose records indicated that they received SP for a much longer period than other cases (up to 490 days). These values were winsorised at 250 days (the 75th percentile plus 1.5 times the IQR) in order to produce descriptive statistics.

We were interested in the number of contacts each individual had with a link worker and the length of time each link worker spent with the client overall (number of contact hours). Contacts could include face-to-face or online meetings, texts or emails, but information on the type of contact was not available. Contacts may lead to individuals being connected with community resources, either through onward referrals or through signposting, which we hereby refer to as receiving an intervention. We used the number of interventions recorded by the link worker. However, a lack of recorded interventions does not necessarily mean that young people did not receive any onward referrals or signposting. Within Joy, interventions were recorded under a separate tab on the client’s page, which was often not completed by link workers who preferred to record details in staff notes instead.

Over 70 intervention types were reported. We classified them into five domains: 1) mental health and well-being support, 2) community activities, 3) practical support, 4) special educational needs and disabilities (SEND) support or 5) other services (online supplemental table S3). Domains were not mutually exclusive, as people were prescribed multiple interventions. Intervention domain was missing for 33% of the recorded interventions.

### Statistical analysis

We first summarised age, referral reasons and referral sources for the full analytical and pre-post samples. Next, we described the SP pathway for the pre-post sample, including number of link worker contacts, contact hours and types of interventions prescribed. We then evaluated SP using a two-tailed paired t-test, assessing whether there was a reduction in SWEMWBS scores from baseline to follow-up. Assumptions of the paired t-test were met, with differences of the paired values normally distributed.

In exploratory supplementary analyses, we tested whether changes in well-being were influenced by age at referral and whether individuals were experiencing depression at baseline. We used a SWEMWBS score of 18 or less to indicate probable clinical depression at baseline. This threshold was developed in adults through comparison to the Patient Health Questionnaire (PHQ-9).^25^ We then used linear regression models testing the association between timepoint and SWEMWBS score, with interactions between timepoint and the two potential effect modifiers in separate models. Cluster-robust SEs accounted for repeated measures within individuals.

## Findings

### Eligible individuals

Among the 770 eligible clients referred to SP, ages ranged from 11 to 25 years (mean=15.4, SD=2.3; online supplemental table S4). Most individuals were referred by their GP (69%) or school (22%), with relatively few referred by other medical services (5%), youth services (3%) or self-referral (1%).

**Figure 2:**
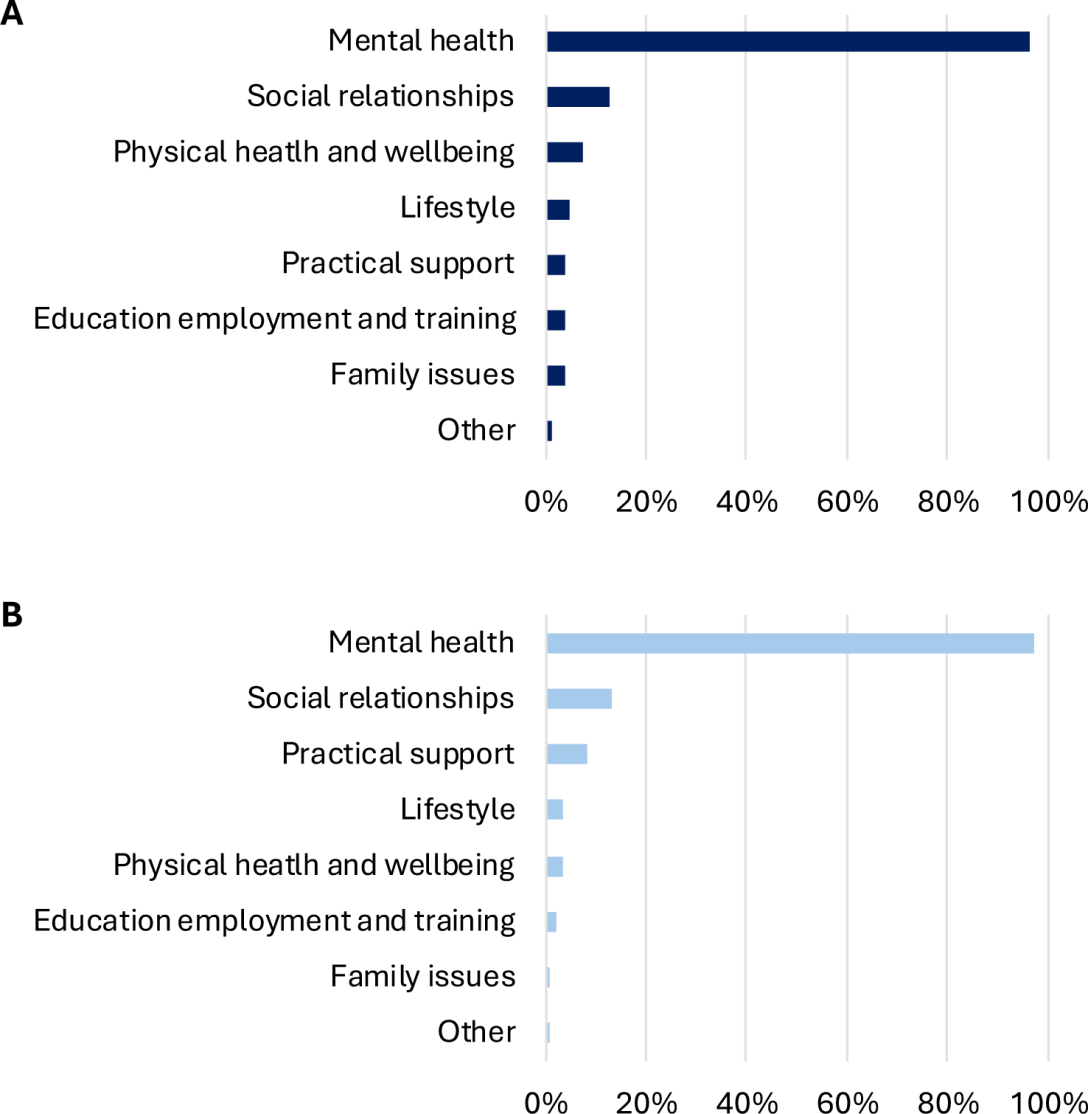
Referral reasons for (A) the analytical sample (n=740; n=30 missing) and (B) the pre-post sample (who were successfully discharged and completed outcome measures; n=192; n=11 missing). Domains were not mutually exclusive, allowing young people to have more than one referral reason.

Overall, 42% of individuals were referred for multiple reasons. Looking at referral reasons separately, mental health was the most common (listed for 97% of people), followed by social relationships (13%) and other reasons (13%; figure 2A). Family issues was the least common domain (4%). Referral rate for practical reasons was similar (4%), but this was never used as the sole referral reason. Of those referred for mental health reasons, 41% also had other referral reasons.

Across all eligible referrals who completed the SWEMWBS at their first session (n=527), the mean score was 18.83 (SD=2.76). Among those referred for their mental health (n=492), the mean SWEMWBS score was 18.84 (SD=2.76). Although the average SWEMWBS score was lower among those who did not have mental health listed as a referral reason (n=15; mean=18.26, SD=2.38), a two-tailed independent samples t-test indicated that this difference was not significant (p=0.422).

Of the 770 eligible CYP, 391 were successfully discharged and 204 were discharged early (as they had declined SP, did not attend or were inappropriate referrals, among other reasons). These two groups had very similar SWEMWBS scores at baseline (online supplemental table S4), although those who were successfully discharged were slightly younger (mean=15.49, SD=2.17) than those discharged early (mean=16.15, SD=2.26). The successfully discharged group were also slightly more likely to have been referred by their GP (74% vs 70%) or school (19% vs 17%), and less likely to be referred by other routes (8% vs 13%), than those who were discharged early.

### Individuals who completed SP

The 203 CYP in our pre-post sample were aged 12–20 (mean=15.2, SD=2.0), with 74% referred by their GP, 22% by school and <5% by other routes. None were self-referrals (online supplemental table S4). Overall, 34% were referred for multiple reasons, with mental health the most common reason (97%), followed by social relationships (13%) and other reasons (8%; figure 2B). Practical support and family issues were least common (<5%). In this subsample, the mean baseline SWEMWBS score was 18.74 (SD=2.55).

#### SP pathways

**Figure 3:**
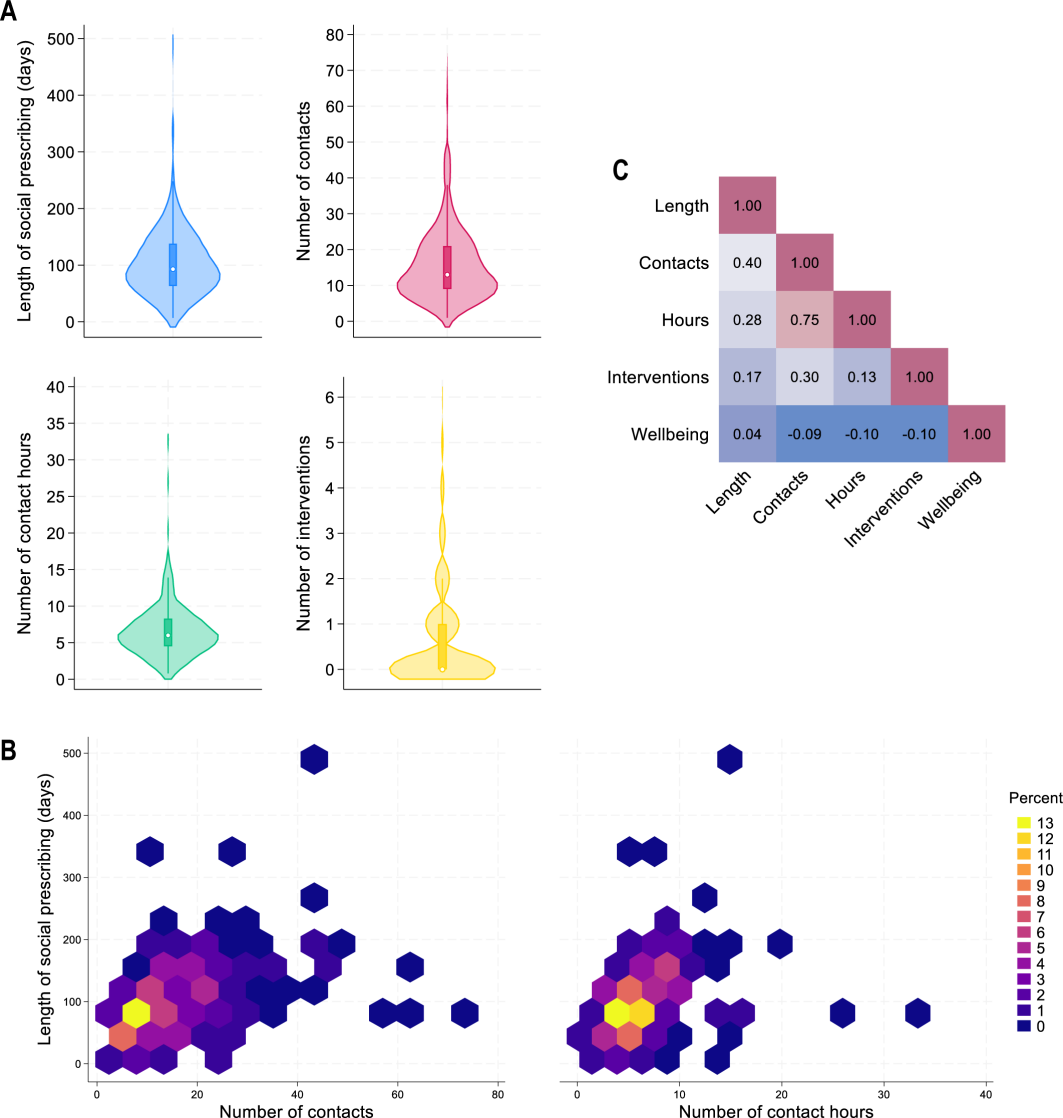
Descriptive statistics for social prescribing pathways in the pre-post sample (n=203). (A) Violin plots showing distribution of the length of social prescribing, number of contacts with a link worker, number of contact hours and number of recorded interventions. (B) Hexagon heat plots showing the distribution of number of contacts and number of contact hours by the length of the social prescribing period. (C) Heat plot of the correlations between social prescribing characteristics and baseline well-being (short Warwick-Edinburgh Mental Wellbeing Scale (SWEMWBS) score). All plots are based on raw data (outliers not winsorised).

In this pre-post sample, link workers recorded between 1 and 72 contacts with clients (mean=16.7, SD=11.8, median=13.0; figure 3A), over a mean of 92.8 days between the first and last contact (SD=60.3, median=88.9, range from 7 to 250 days). Contact hours ranged from 0.8 to 32.7 hours, with a mean of 6.7 hours (SD=4.0, median=6.0). Although there was a lot of variability, the most common SP pathway was to receive SP for around 90 days, with 10–15 link worker contacts (which could include texts and emails as well as meetings) and 6 contact hours (figure 3B).

The length of the SP period was moderately positively correlated with the number of contacts (r=0.40; figure 3C), but less strongly related to the number of contact hours (r=0.28). The number of contacts and contact hours were strongly correlated (r=0.75). But these SP characteristics were only weakly related to young people’s baseline well-being (r=−0.10 to 0.04). Looking at referral reasons, those with a single referral reason had more contacts (mean=17.6, SD=12.0) and more contact hours (mean=7.1, SD=3.7) than those with multiple referral reasons (contacts=14.2, SD=11.3; hours=5.8, SD=4.4).

**Figure 4:**
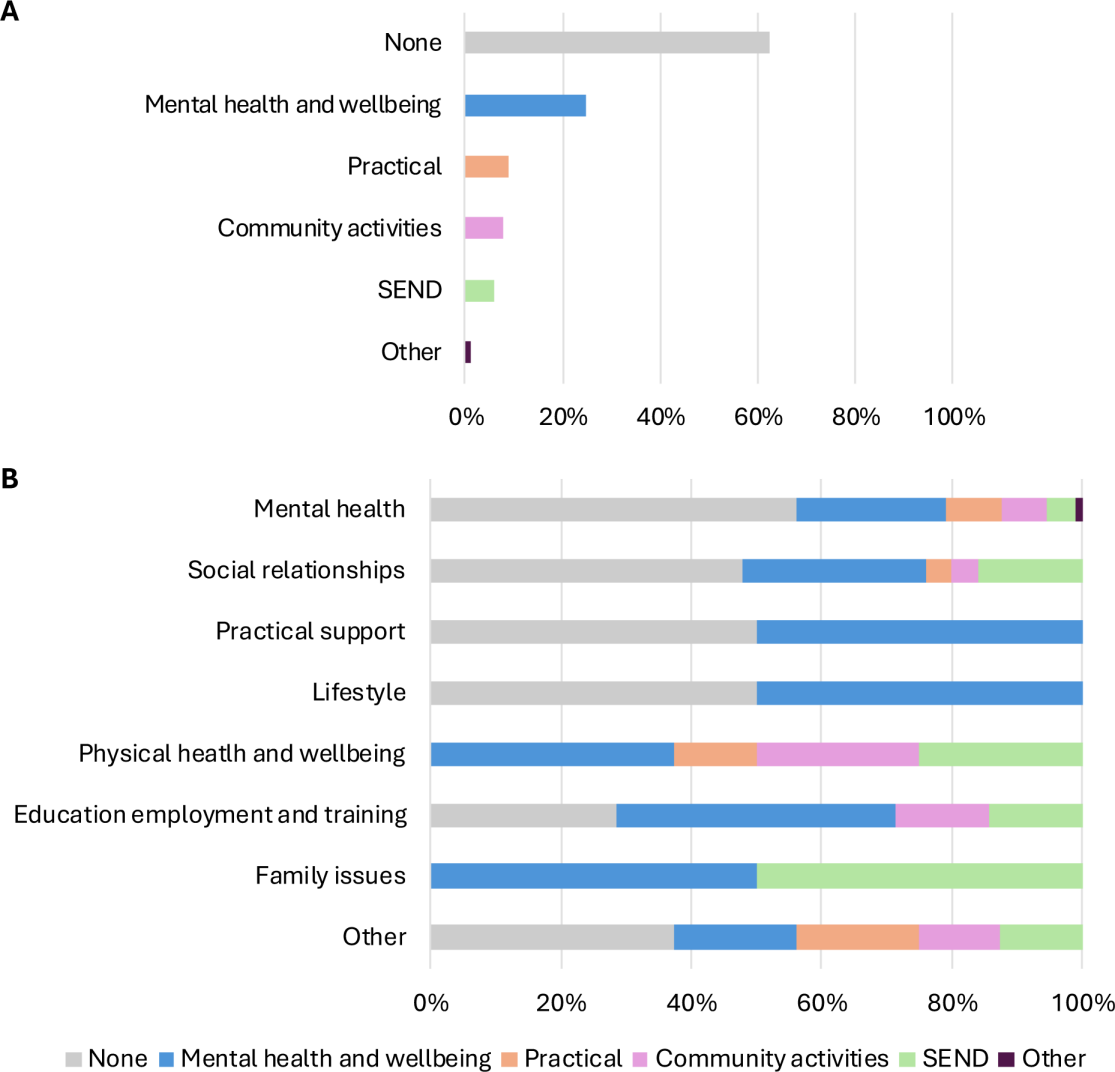
Intervention domains for (A) those who were successfully discharged and completed outcome measures (the pre-post sample; n=203) and (B) for the same group, shown separately according to their reasons for referral (group size differed across referral reasons). Domains were not mutually exclusive, as young people may have been referred/signposted to more than one intervention. SEND, special educational needs and disabilities.

No intervention was recorded for 63% of CYP (figure 4A). For those with interventions recorded (n=76), number of interventions ranged from 1 to 6 (mean=1.9, SD=1.2). Intervention domain was reported for 68% of all recorded interventions, with mental health and well-being support most common (66%), followed by practical support (24%) and community activities (21%). SEND support (16%) and other services (3%) were least common.

We then explored whether prescribed interventions corresponded to referral reasons (figure 4B). Of those referred because of mental health, 56% had no intervention recorded. Similar proportions of those referred for support with lifestyle (50%), practical issues (50%) and social relationships (48%) had no intervention recorded. In contrast, all of those referred for family issues and physical health had an intervention recorded, most frequently a mental health and well-being intervention (family issues=50%, physical health=38%). However, these proportions were likely influenced by small and differing group sizes across referral reasons (n=2–187).

#### Efficacy of SP

A paired t-test indicated that, following SP, SWEMWBS scores improved by an average of 3.72 points (t(202)=17.50, mean diff=3.72, 95% CI 3.30 to 4.14, p<0.001, d=1.23). This is equivalent to a 20% relative increase in well-being from the first to final session. At the final session before discharge, the mean SWEMWBS score was 22.46 (SD=3.28).

In exploratory analyses, there was evidence that the effect of SP differed according to well-being at baseline (online supplemental figure S1B), with a larger increase in well-being among those who met the criteria for probable depression at baseline compared with those who did not (coef=1.31, 95% CI 0.49 to 2.13, p=0.002). However, SWEMWBS scores still improved over time in both subgroups. There was no evidence for effect modification by age at referral (online supplemental figure S1A and table S5).

## Discussion

We explored the feasibility of using routine data from the Joy platform^24^ to evaluate an SP service in North East England. Over 18 months from 2023 to 2024, 770 eligible 11 to 25 year olds were referred and 391 were successfully discharged. Only 203 could be included in analyses of the impact of SP on well-being, largely due to missing data on well-being. Data quality precluded detailed analyses of inequalities in access to SP and the care pathway. However, we saw an increase in well-being from the first to the final session of SP. Exploratory analyses suggested that this increase differed according to the initial levels of well-being.

### Data quality

Our first objective was to understand data quality. Missing data was the largest issue. Only 6–8% of CYP had gender/ethnicity recorded, so these could not be used. Just 57% of those successfully discharged had complete well-being measures, increasing the risk of selection bias. Only 36% of CYP who received SP had an intervention recorded, but no recorded interventions did not mean that no intervention was prescribed, so analyses using the number of interventions may not be valid. A lack of detail in some fields also presented challenges. We could not determine why some CYP were inappropriate referrals or discharged early. Understanding how many SP sessions each person received was difficult as we could not distinguish between contacts to arrange appointments and sessions. Additionally, link workers could not record re-referrals, meaning some cases appeared to be open for a long period, with very high numbers of sessions and contact hours, which may have resulted from multiple referrals.

Data limitations create issues for policy, practice and research. The NHS England SP Information Standard recommends that patient demographics, needs and concerns, and support offered (including referrals) are recorded, alongside outcomes measured using the Office for National Statistics well-being measures (ONS4) or WEMWBS.^28^ The National Academy for SP (NASP) similarly recommends that interventions are recorded and a validated health/well-being questionnaire used to allow process and impact evaluation.^29^ In conversations with the service that provided these data, it emerged that gender and ethnicity were not compulsory fields in Joy, which likely contributed to high levels of missingness. Similarly, fields for recording interventions were not compulsory, meaning this information was often limited to staff notes that could not be examined in this evaluation. In a 2025 survey of UK link workers, 24% said they never recorded outcome measures.^17^ Barriers to recording outcomes included a lack of appropriate sensitive and standard measures, little guidance on who should record outcomes or when and how to use measures, fragmented systems and time constraints.^17^

To improve practice, we recommend further collaboration between platform providers and SP services. Services should be able to mandate fields, record why individuals are not eligible for SP or are discharged, identify re-referrals and distinguish between contact types. NHS England recommends that SP systems link information from the national database of NHS patient demographics. Platforms currently differ in the functions offered, so SP services may wish to consider whether these are possible before choosing a platform. For those delivering SP, better digital tools, more training, standardised guidelines, reminders and designated time for updating client records are needed.^17^ Further discussions may be needed around the utility of outcome measurement, particularly as 74% of link workers in the previous survey did not know whether outcomes were used to influence investment decisions.^17^

### Individual characteristics and the SP pathway

Our second objective was to explore access to and characteristics of SP for CYP. This was challenging due to the data limitations. Most CYP were referred by their GP or school. Referral reasons were very different to previous findings in adults, a third of whom were referred for mental health (vs 97% of CYP) and a quarter for practical support (vs 4% of CYP).^2^ Although the CYP service did not specialise in mental health, service staff believe that high levels of referrals due to mental health reflected the deprivation, rurality and lack of timely mental health support within the county. In our pre-post sample, the most common pathway was to receive SP for around 90 days, with 10–15 link worker contacts and 6 contact hours. However, there was wide variation in the SP period (up to 490 days), contacts (up to 72) and hours (up to 33). Service staff reported that some of the extreme values may be due to safeguarding concerns or complex needs, where SP likely was not appropriate but CYP were not discharged so that staff could ensure they were seen by appropriate services (many of which had significant waiting lists). Some of these CYP were offered a listening service until they could be seen by other services, and link workers were able to advocate for the CYP if they could not do this themselves. However, most long SP periods with many contacts were likely driven by re-referrals.

For the minority of CYP with intervention domains recorded, the most common were mental health and well-being support (66%), practical support (24%) and community activities (21%). Prescribed interventions did not match referral reasons well. Although it is hard to draw conclusions given the proportion of missing data, some of this disparity may be due to the explicit focus on what matters to individuals in SP. This may mean that interventions are well aligned to CYP’s own perceived needs, which are not necessarily why they were referred. Additionally, as baseline well-being did not differ across those with and without mental health listed as a referral reason, the recorded reasons for referral may not accurately represent CYP’s current state. The low proportion of self-referrals (1%) may also contribute to this finding.

### Efficacy of SP

Our final objective was to assess the impact of SP on well-being. We found an average relative increase in well-being of 20% from the first to the last SP session. This corresponded to statistics provided to the SP service in a Joy monitoring dashboard. While clinically meaningful,^25^ this is <4 SWEMWBS points, which sounds less significant than a 20% increase. It is thus important to present absolute alongside relative outcomes, and for dashboards to describe how statistics are calculated, including the underlying number of clients. Until then, practitioners cannot rely on dashboards to make informed decisions about services.

Despite restricting the pre-post sample to those with complete data, and thus limiting generalisability, our findings are consistent with other evaluations. Uncontrolled studies among CYP have found similar pre-post increases in well-being in analyses of the SWEMWBS,^8^ ONS4^8 13^ and descriptive statistics.^9 10 12^ National studies including all ages have found similar evidence, with analyses of records from Elemental showing a 3.31-point increase in SWEMWBS score^18^ and an evaluation of green SP (focused on nature-based activities) finding small but robust increases on the ONS4.^30^ Our exploratory analyses indicated that those with lower well-being before starting SP experienced larger increases in well-being following SP, which is also consistent with other evidence.^8 9^ This could be because these CYP had the greatest room for improvement or because SP has greater impact for those with the worst mental health (who are still eligible and able to engage).

### Strengths and limitations

This study had several strengths, including being the first to use routine data to evaluate CYP SP. This enabled measurement of real-world heterogeneity and identification of strategies to improve data collection and quality. Well-being was measured with the SWEMWBS, which has been validated for this population^27^ and used in many evaluations of SP.^3 18^ However, 43% of CYP successfully discharged from SP had missing well-being data. Although the pre-post subsample was comparable to those referred to SP in terms of average age, referral routes/reasons and well-being, this may have introduced selection bias in unmeasured characteristics. Additionally, missing outcome data limited the size of our pre-post sample, which reduced power for exploratory analyses of effect modification. We could not use multiple imputation because of the lack of demographic information. However, service staff reported that outcome data were often missing because, once CYP felt their goals had been met, many preferred to be discharged instead of attending further appointments to complete the well-being measure. This could mean that we underestimated the effect of SP on well-being, as those with improved well-being may be less likely to complete outcome measures.

Another major limitation is the lack of control group, as all CYP received SP. Our findings could thus reflect regression to the mean, meaning low well-being scores may appear to improve without any intervention. Given that no control group can be derived from SP records, future research should consider using synthetic controls or digital twins. However, detailed individual-level demographic and socioeconomic information is needed for these approaches. It is thus not yet feasible to create matched controls. This lack of information also precludes further analyses of inequalities in access to and engagement with SP. Furthermore, data quality meant that analyses of the number of interventions may reflect link workers’ recording practices rather than actual interventions received. Finally, we had planned to test whether changes in well-being differed according to the SP received, including number of contacts, contact time and number of interventions. However, this was not feasible in these pre-post analyses, as the SP pathway may be influenced by baseline well-being. Indeed, link workers reported that they may give less practical support to those with better well-being at baseline, as they are already able to engage in activities. Contacts and number of interventions could thus be mediators on the causal pathway, instead of effect modifiers. Future research should explore whether the amount of SP received influences outcomes.

## Clinical implications

We have identified the challenges of using routine data to describe and evaluate SP for CYP. Despite numerous data limitations, we found that SP, as currently implemented in one area, is associated with improvements in CYP well-being. We identified key priorities for enhancing data quality. Bespoke electronic records providers should consider compulsory recording of demographics, the ability to identify re-referrals and clear recording of SP sessions. To support those working in SP, we recommend more training, better tools for recording outcomes, standardised guidelines and processes, resources to help demonstrate the need to measure outcomes to CYP, and designated time for updating records. Finally, it is vital that policymakers and researchers continue to advocate for high quality data, particularly outcome measures.

## Supplementary material

10.1136/bmjment-2025-302442 online supplemental file 1

## Data Availability

No data are available.
