# Supplementary material for: Using electronic health records to evaluate a children and young people’s social prescribing service: challenges and implications for research and practice
Source: BMJ Ment Health. 2026 Apr 24;29(1):e302442. doi: 10.1136/bmjment-2025-302442 (PMC13110528; doi:10.1136/bmjment-2025-302442)
Supplement: online supplemental file 1 [file bmjment-29-1-s001.docx]

Supplementary Materials

**Table S1.** Missing data on study variables.

| **Measure** | **Analytical sample**  (n=770) | **Pre-post sample** (n=203) |
| --- | --- | --- |
|  | N (%) missing | |
| Age | 0 | 0 |
| Gender | 711 (92%) | 187 (92%) |
| Ethnicity | 721 (94%) | 190 (94%) |
| Referral source | 1 (<1%) | 0 |
| Referral reason | 30 (4%) | 11 (5%) |
| SP length | 548 (71%) | 0 |
| Number of contacts | 0 | 0 |
| Number of contact hours | 0 | 0 |
| Number of interventions | 0 | 0 |
| Intervention domain  *(proportion of prescribed  interventions)* | 112 (33%) | 46 (32%) |
| Wellbeing (SWEMWBS) |  |  |
| Baseline | 243 (32%) | 0 |
| Follow-up | 511 (66%) | 0 |

*Note.* Dashes indicate no missing data.

**Table S2.** Examples of reasons for referral to social prescribing, which were recorded using free text.

| **Domain** | **Examples** |
| --- | --- |
| Mental health | anger and frustration; anxiety; bereavement; coping with change; depression; emotional wellbeing; feeling low; feeling unsafe; lack of motivation; low mood; panic attacks; previous self-harm, self-esteem; social anxiety; stress; suicidal ideation; traumatic life events |
| Physical health and wellbeing | ADHD; autism; coeliac disease; cognitive disorder; endometriosis; health and wellbeing support; high body weight; managing a long-term condition; neurodiversity; physical health; sexual health; Tourette’s |
| Social relationships | concerns around socialisation; friends; loneliness/isolation; relationships; sex and relationships; struggling to make friends |
| Family issues | family difficulties; family problems; issues with family; relationship with family |
| Lifestyle | finding things to enjoy; problems with sleep; relationship with food; sedentary lifestyle; substance misuse; weight management |
| Education employment and training | education; employment; not supported at school; school attendance; school life; work/education; work/training/education |
| Practical support | caring responsibilities; day-to-day helping hand; finances; housing problem; money/benefits |
| Other | bullying; connecting to services; domestic abuse in the home; gender/sexual identity; in foster care; parental split; problems at home; transitioning gender; victim of abuse |

**Table S3.** Examples of interventions that people were referred to, which were recorded using free text.

| **Domain** | **Examples** |
| --- | --- |
| Mental health and wellbeing support | Bereavement Counselling; Calm Harm app; CAMHS; Community paediatric diabetes service; Headspace; IAPT; OCD Action; Samaritans; YoungMinds |
| Community activities | boxing club; disability football group; drama classes; horse riding; library reading group; mums and friends group; Scouts; tennis club; youth action group |
| Practical support | Citizens Advice; domestic abuse support services; food bank; safeguarding children partnership; weight management service |
| Special educational needs and disabilities (SEND) support | Early Help support; parent carer forum; SENDIASS; sensory processing hub |
| Other | meditation; mindfulness; sleep; small locally focused charity |

**Table S4.** Individual characteristics for the full analytical sample and relevant subsamples, alongside a description of the social prescribing pathway for the pre-post sample.

|  | **Analytical  sample** (n=770) | **Successful  discharge** (n=391) | **Early discharge*** (n=204) | **Pre-post sample** (n=203) |
| --- | --- | --- | --- | --- |
| **Individual characteristics** |  |  |  |  |
| Age (years) | 15.38 (2.25) | 15.49 (2.17) | 16.15 (2.26) | 15.24 (1.98) |
| Referral source |  |  |  |  |
| GP | 69% | 74% | 70% | 74% |
| School | 22% | 19% | 17% | 22% |
| Other medical | 5% | 8% | 13% | <5% |
| Youth services | 3% |  |  |  |
| Self-referral | 1% |  |  |  |
| Baseline wellbeing (SWEMWBS) | 18.83 (2.76) | 18.85 (2.84) | 18.69 (2.63) | 18.74 (2.55) |
| **Social prescribing pathway** |  |  |  |  |
| Social prescribing length (days) | - | - | - | 104.66 (61.72) |
| Number of contacts | - | - | - | 16.71 (11.83) |
| Number of contact hours | - | - | - | 6.67 (3.97) |
| Number of interventions | - | - | - | 0.70 (1.17) |

*Note.* * Early discharge includes cases where the client declined SP or did not attend sessions, inappropriate referrals, and discharged for other reasons (usually inappropriate for SP).

**Table S5.** Linear regression models testing whether the association between time and wellbeing score is modified by individual characteristics in the pre-post sample (n=203).

| **Effect modifier** | **Coefficient (95% CI)** | **p value** |
| --- | --- | --- |
| Baseline wellbeing  (ref: no probable depression) | 1.31 (0.49, 2.13) | 0.002 |
| Age  (continuous years) | 0.14 (-0.08, 0.36) | 0.220 |

*Note.* Coefficients shown are for the interaction between the effect modifier and time. Each row in the table shows results from separate models.

**Figure S1.** Exploratory analyses testing whether the change in wellbeing over time differed according to individual characteristics in the pre-post sample (n=203). Estimates from linear regression models with an interaction between timepoint and A) age in years (analysed continuously but model estimates shown for ages 12, 16, and 20 years) and B) whether individuals met the criteria for probable clinical depression at baseline. Confidence intervals use cluster-robust standard errors, which account for the repeated measures within individuals.
